# Intravenous Contrast Agent in Abdominal CT: Is It Really Needed to Identify the Cause of Bowel Obstruction? Proof of Concept

**DOI:** 10.1155/2019/2350948

**Published:** 2019-09-05

**Authors:** Federica Vernuccio, Dario Picone, Gregorio Scerrino, Massimo Midiri, Giuseppe Lo Re, Roberto Lagalla, Giuseppe Salvaggio

**Affiliations:** ^1^Department ProMISE (Department of Health Promotion, Mother and Child Care, Internal Medicine and Medical Specialties), University Hospital of Palermo, Piazza delle Cliniche, 2, 90127 Palermo, Italy; ^2^University Paris Diderot, Sorbonne Paris Cité, Paris, France; ^3^I.R.C.C.S. Centro Neurolesi Bonino Pulejo, Contrada Casazza, SS113, 98124 Messina, Italy; ^4^Department of Biopathology and Medical Biotechnologies, University Hospital of Palermo, Via del Vespro 129, 90127 Palermo, Italy; ^5^Unit of General and Emergency Surgery, Department of Surgical, Oncological and Oral Sciences, University of Palermo, 90127 Palermo, Italy

## Abstract

**Background:**

To compare sensitivity of unenhanced computed tomography (CT) and contrast-enhanced CT for the identification of the etiology of bowel obstruction.

**Materials and Methods:**

We retrospectively evaluated abdominal CT scans of patients operated for bowel obstruction from March 2013 to October 2017. Two radiologists evaluated CT scans before and after contrast agent in two reading sessions. Then, we calculated sensitivity of CT in the diagnosis of bowel obstruction and determined in which cases the etiology of bowel obstruction was detected on both unenhanced and enhanced CT or on enhanced CT only. The reference standard was defined as the final diagnosis obtained after surgery.

**Results:**

Eighteen patients (mean age 72 ± 15 years, age range 37-88 years) were included in the study. Sensitivity of unenhanced CT and enhanced CT was not significantly different in either small bowel obstruction (64%, 7/11 patients vs. 73%, 8/11 patients; *P* = 0.6547) or large bowel obstruction (71%, 5/7 patients vs. 100%, 7/7 patients; *P* = 0.1410). Adhesions were identified on unenhanced CT as the etiology of small bowel obstruction in 80% (4/5) of patients. Tumors were identified on unenhanced CT as the etiology of large bowel obstruction in 67% (4/6) of patients.

**Conclusion:**

In the diagnosis of small bowel obstruction due to adhesions with normal bowel wall thickening and when a neoplasm is identified as the etiology of large bowel obstruction on unenhanced CT, an intravenous contrast agent may be avoided for the identification of the etiology. In remaining cases, contrast agent is still recommended.

## 1. Introduction

Bowel obstruction may be the result of a mechanical obstacle or a failure of the bowel to move properly (i.e., paralytic ileus). Mechanical obstruction accounts for approximately 15%-20% of admissions to the emergency department overall for acute abdomen [1], and it is the most common cause of acute abdominal pain in elderly patients [2, 3]. Small bowel obstruction is four to five times more common than large bowel obstruction [4], and the potential etiology differs substantially in small compared to large bowel obstruction [4]. In patients with suspected bowel obstruction, the main tasks of radiologists include the determination of transition point and etiology, as well as the presence or absence of complications such as ischemia or perforation. Computer tomography (CT) allows to differentiate mechanical bowel obstruction from paralytic ileus as well as to identify the transition point and, eventually, bowel ischemia in mechanical obstruction [5, 6].

CT protocol in patients with suspected bowel obstruction has been studied for years. It is well known that the oral administration of a high-attenuation contrast agent is no longer recommended for this diagnosis [7]. While there is a consensus on contrast-enhanced CT as the first-line examination for abdominal emergencies, the value of unenhanced CT - that is yet performed in many institutions - is more controversial [2, 3, 8–13]. In a patient with acute abdominal pain, many centers use a CT protocol with a single portal-venous phase acquisition as indicated by ACR appropriateness criteria—without a precontrast scan—assuming that the value of unenhanced images is limited [8, 9]. Intravenous contrast agent is beneficial to assess complications including inflammation and ischemia; specifically, the diagnosis of bowel ischemia requires contrast agent administration mandatory [2]. Despite abdominal CT after intravenous contrast agent injection is the radiologic procedure with the highest diagnostic performance rating according to ACR guidelines [8, 9], there are many drawbacks related to the use of intravenous iodinated contrast agents. Therefore, the use of intravenous iodinated contrast agents for acute abdomen has been recently questioned in specific settings, particularly in elderly patients and in patients with nontraumatic acute surgical abdomen [2, 3, 10].

Barat et al. [2] recently demonstrated comparable diagnostic accuracy of unenhanced and enhanced CT (64%-68% vs. 68%-71%, respectively) in elderly patients presenting to the emergency department with acute abdominal pain, including those with mechanical bowel obstruction. Considering that mean age of surgical patients with bowel obstruction has gradually increased over centuries by more than 20 years [14], it is important to investigate the benefit of intravenous contrast agent for the diagnosis of the etiology of bowel obstruction. An intravenous contrast agent may worsen an eventually impaired renal function—which is not uncommon in elderly patients—leading to contrast-induced nephropathy in up to 5% of patients [15]; in addition, it may be responsible for adverse drug reactions, and it is costly and time-consuming. Specifically, despite new assay methods, obtaining baseline serum creatinine level remains time-consuming and may delay treatment. Therefore, it is still debated whether the administration of an intravenous iodinated contrast agent in bowel obstruction should be considered mandatory. Our hypothesis is that the unenhanced CT scan should be sufficient to identify the cause of obstruction in most cases. Therefore, the purpose of this study was to compare diagnostic accuracy of unenhanced CT and contrast-enhanced CT scan for the identification of the etiology of bowel obstruction.

## 2. Materials and Methods

This retrospective, Health Insurance Portability and Accountability Act-compliant study was approved by the Institutional Review Board of our hospital, and a waiver of informed consent was obtained.

### 2.1. Study Cohort

**Figure 1:**
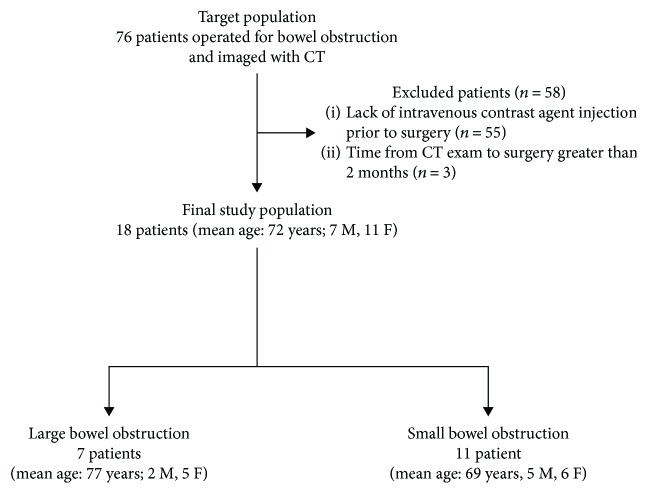
Flowchart shows study enrollment based on recommended Standards for Reporting of Diagnostic Performance criteria.

Figure 1 portrays the patient accrual flowchart following Strengthening the Reporting of Observational Studies in Epidemiology (STROBE) initiative [16]. We retrospectively searched the electronic medical record database at our Institution, a tertiary academic center, for consecutive patients operated because of bowel obstruction and imaged with CT between March 1, 2013, and October 31, 2017. The decision to treat patients with bowel obstruction was made as a consensus by a panel composed of an emergency physician, an abdominal surgeon, and a radiologist.

Our search yielded an initial target population of 76 consecutive patients who were considered eligible for inclusion in the study. Subjects were excluded for (a) lack of intravenous contrast agent administration prior to surgery (*n* = 55) and (b) time from CT exam to surgery greater than 2 months (*n* = 3). The final population consisted of 18 patients (mean age 72 ± 15 years, age range 37-88 years), including 7 men (mean age 74 ± 12 years, age range 59-88 years) and 11 women (70 ± 17 years, range, 37–87 years).

### 2.2. CT Imaging Technique

Dynamic contrast-enhanced abdominal CT exams had been performed using a 16-row multislice CT (G.E., Milwaukee, WI). Acquisition parameters were as follows: 120 kVp; 200-400 mA; 16 × 1.25 mm mm detector configuration; section thickness/interval, 3.75/3.75 mm; and 0.5-second rotation time. The scanner used automatic exposure control. After unenhanced imaging, patients received a weight-based dose of iodinated contrast material (volume: 80-130 ml; iodine concentration: 320, 350, 370, and 400 mg iodine/ml) that was injected through a user-programmable single-head injection system, through an antecubital vein at a rate of 2.5-3.5 ml/s, according to the venous access available. CT protocol included unenhanced and portal venous phases. Scan delay was obtained using bolus tracking technique with the region of interest placed in the aorta at the level of the diaphragm. The arterial and/or the delayed (2-3 minutes) phases were additionally acquired when active bleeding or vascular compromise of the bowel was suspected. In none of the patients, an oral contrast agent was administered.

### 2.3. CT Imaging Analysis

CT exams were reviewed by two radiologists first separately and then in consensus, using a clinical picture archiving and communication system (AGFA Impax, Mortsel, Belgium). The radiologists were blinded to the purpose and the design of the study, the clinical history, clinical report, and surgery findings.

To minimize recall bias, all technical and personal data were removed from the images and datasets were randomized prior to all reading sessions. Two reading sessions separated by 4 weeks were performed—one session showing the unenhanced CT images alone and the other session showing both the unenhanced and the contrast-enhanced images. During each reading session, readers were asked to identify the etiology and the level of the obstruction (i.e., small or large bowel obstruction). The order by which unenhanced only and unenhanced plus enhanced images were presented to the readers during the two reading sessions was randomly assigned.

The surgical diagnosis of the etiology was considered the reference standard.

### 2.4. Statistical Analysis

Data were collected in an Excel database (Microsoft Corporation, Redmond, WA, USA), and statistical analysis was performed using GraphPad (San Diego, CA, USA) software. The sensitivity and specificity of unenhanced CT and enhanced CT were calculated. Comparisons between groups were performed using the McNemar test, Fisher's exact test, or chi-square test, as appropriate. A *P* value < 0.05 was considered statistically significant. Cohen's kappa statistics were applied for measuring agreement between two readers.

## 3. Results

Table 1 summarizes the etiology of bowel obstruction in the patient study cohort. Small and large bowel obstructions were encountered in 11 of 18 (61%) patients and 7 of 18 (39%) patients, respectively. Sensitivity of unenhanced CT and enhanced CT in the assessment of etiology of bowel obstruction was 67% (12 of 18 patients) and 78% (14 of 18 patients), respectively (*P* = 0.4795). An interreader agreement in the recognition of etiology was very high (Cohen's kappa = 0.95).

**Figure 2:**
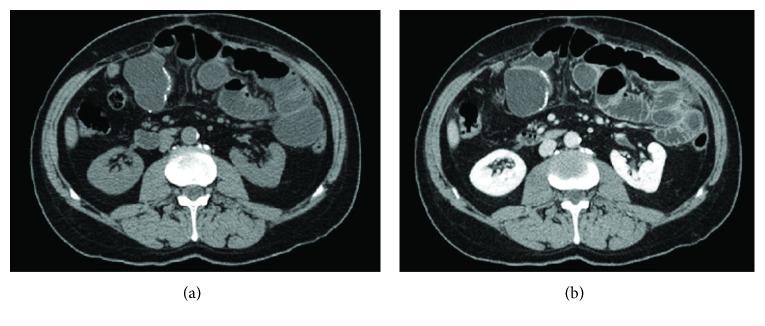
Axial CT scan of a 64-year-old man previously operated for internal hernia with resection of some ileal loops. Unenhanced phase (a) showed dilatation of the duodenal, jejunal, and proximal ileal loops with the transition point at the level of the ileo-ileal anastomosis; the etiology of the small bowel obstruction was correctly identified as adhesions. Post contrast image in the venous phase (b) showed normal enhancement of the bowel loops.

**Figure 3:**
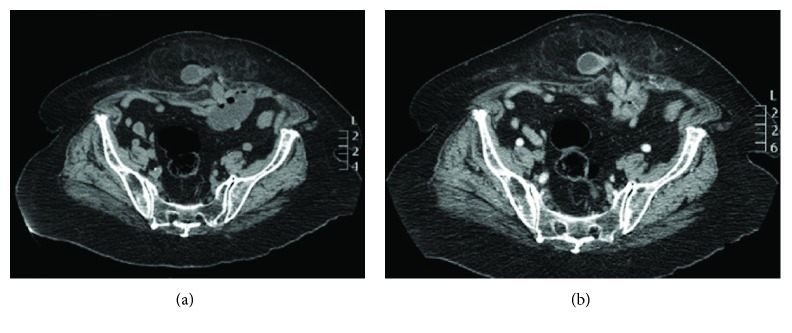
Axial CT scan of an 85-year-old female with abdominal pain. Unenhanced CT scan (a) shows an abdominal incisional hernia with protrusion of omental fat and a small bowel loop, the dilated afferent small bowel loop, and the collapsed efferent loop in the transition zone. Post contrast image in the portal venous phase (b) show normal enhancement of the small bowel loop within the hernia. However, the detection of the etiology of the bowel obstruction was already made on unenhanced CT.

**Figure 4:**
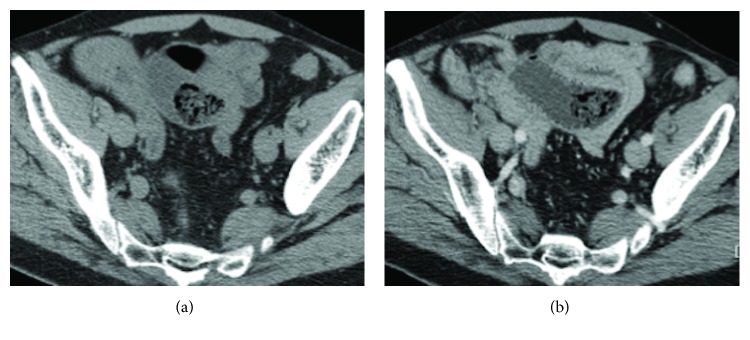
Axial CT scan of a 60-year-old man previously operated of cholecystectomy and splenectomy. Unenhanced phase (a) shows dilatation of the jejunal loops, but a neoplastic origin could not be ruled out. Post contrast image in the portal venous phase (b) shows absence of pathologic enhancement of the bowel wall, and the final diagnosis of small bowel obstruction due to adhesions was made.

The most common etiologies of small bowel obstruction were adhesions in 5 of 11 (45%) patients (Figure 2), incisional hernia in 2 (18%) patients (Figure 3), and volvulus in 2 (18%) patients. In small bowel obstruction, sensitivity of unenhanced CT (64%, 7 of 11 patients) and enhanced CT (73%, 8 of 11 patients) was not significantly different in the identification of the etiology (*P* = 0.6547). Adhesions were correctly identified on 6 unenhanced CT as the etiology of small bowel obstruction in 80% (4/5) of patients with adhesions at surgery. In 3 patients with small bowel obstruction, etiology was missed at both unenhanced and enhanced CT including one patient with volvulus, one with adhesions related to chronic appendicitis and ileal tumor, and one patient with infiltrating retroperitoneal neoplasm that was misdiagnosed on CT as a duodenal tumor. In one patient with adhesions, the readers were unsure of the diagnosis on unenhanced CT and a contrast agent was deemed necessary to rule out tumors (Figure 4).

The most common etiologies of large bowel obstruction were tumors (86%, 6 of 7 patients). In large bowel obstruction, sensitivity of unenhanced CT (71%, 5 of 7 patients) and enhanced CT (100%, 7 of 7 patients) was not significantly different in the identification of the etiology (*P* = 0.1410). Tumors were correctly identified on unenhanced CT as the etiology of large bowel obstruction in 67% (4/6) of patients with tumors at surgery. In 2 patients, the diagnosis was uncertain on unenhanced CT, and a neoplasm was diagnosed after contrast agent administration.

## 4. Discussion

Our preliminary study suggests that sensitivity of unenhanced CT is not significantly improved by the use of an enhanced phase for the identification of the etiology of small and large bowel obstructions compared to unenhanced CT alone. Our study is in agreement with Atri et al. [17] who demonstrated no significant statistical difference between unenhanced and contrast enhanced CT in the detection of the etiology (61.5–69% vs. 69–73.1%, respectively) of small bowel obstruction. However, while the study of Atri et al. [17] was limited to small bowel obstruction, in our study, we demonstrate that unenhanced CT alone might be sufficient for the detection of the etiology also in large bowel obstruction with a sensitivity of 71%. In our experience, unenhanced CT in patients with large bowel obstruction might have a limited role when the etiology is a small tumor and indirect signs—i.e., fat stranding and enlarged pericolic nodes—are lacking.

An interesting finding of our study is that in 80% of our patients with small bowel obstruction due to adhesion bands and in 67% of patients with large bowel obstruction due to tumors, the etiology was already correctly identified on unenhanced CT. Adhesion bands may be encountered as a cause of small bowel obstruction in up to 75% of cases [18, 19], while they are considered a very rare cause of large bowel obstruction [20]. In agreement with prior studies [18–20], the incidence of adhesion bands as the cause of bowel obstruction was also higher in small bowel obstruction (45%) compared to large bowel obstruction (14%) in our patient cohort. CT diagnosis of adhesions is based on an abrupt change in bowel caliber with a focal region of beaklike at the transition point involving the area of previous surgical intervention in the absence of any other possible causes of obstruction [19]. Based on our preliminary findings and on the recent literature [2, 3], we suggest to perform an unenhanced CT scan at first, in adult patients with acute abdominal and referred for suspected bowel obstruction, unless there is suspicion of complications (i.e., bowel ischemia). Intravenous contrast agents have been associated with a wide range of possible side effects, from a mild inconvenience, such as itching, to life-threatening emergencies (i.e., cardiac arrest) [21]. In addition to acute reactions which occur within 1 hour of contrast agent administration, late or very late adverse reactions may occur and their incidence is difficult to be known exactly [22, 23]. Among these, thyrotoxicosis may occur in at-risk patients including those with multinodular goiter and thyroid autonomy, especially if they are elderly and/or live in an area of dietary iodine deficiency, patients with impaired renal function (i.e., predialysis CKD and end-stage renal disease), or a history of thyroid dysfunction [22, 23]. Finally, post contrast acute kidney injury—which was overstated in the past—may be related to some patient-related factors such as old age, classic cardiovascular and metabolic risk factors, malignancy, inflammation, bleeding, anaemia, and hyperuricaemia; moreover, the effect of two or more risk factors is additive and increases its risk [21, 24]. Considering that bowel obstruction is more commonly diagnosed in older patients [14] and that older patients may have those comorbidities considered risk factors for contrast adverse effects more commonly than young patients, the acquisition of unenhanced CT scan as the first approach in elderly patients with suspected bowel obstruction seems reasonable. This should also allow to avoid delays in obtaining creatininemia laboratory results in overcrowded emergency department.

In agreement with prior studies [2, 17], interobserver agreement for the diagnosis of the etiology of bowel obstruction at unenhanced and enhanced CT in our study was good (95%). A slight level of disagreement was observed for difficult diagnoses—i.e., small tumors in the large bowel and volvulus—which could be explained by the higher experience of one of the two readers. Both the readers missed, misdiagnosed, or were very uncertain of the etiology in 4 patients with uncommon causes of bowel obstruction such as ileal tumor and retroperitoneal infiltrating tumor. Given their low frequency, these etiologies are less likely to be considered within the differential diagnosis by the radiologists independently from intravenous contrast agent administration.

In addition to its retrospective design, several limitations of our study deserve attention. First, we excluded patients operated for bowel obstruction and imaged only with unenhanced CT. Although this reflected the aim of our study—which was the comparison of sensitivity of unenhanced and enhanced CT—this might have underestimated sensitivity of unenhanced CT in those cases where a contrast agent was deemed not necessary by the radiologist. Another important limitation is the small number of patients included in our study (*n* = 18). This is due to the strict inclusion and exclusion criteria which required both pathology proof and intravenous contrast agent administration. Moreover, we acknowledge the single-center experience and the use of only a 16-slice CT scanner as potential limitations that limit generalizability of our results. Future multicenter studies in larger patient cohorts are warranted to validate our preliminary findings.

In conclusion, our preliminary data suggest that in regard to the identification of the etiology of bowel obstruction, when small bowel obstruction is likely due to adhesions and bowel wall thickening is normal on unenhanced CT and when a neoplasm is clearly identified on unenhanced CT as the cause of large bowel obstruction, intravenous contrast agent may be potentially avoided. In the remaining cases, intravenous contrast administration is still recommended.

## Figures and Tables

**Table 1:** Etiology and location of bowel obstruction in the study population.

	Small bowel obstruction 11 patients	Large bowel obstruction 7 patients
Etiology, *n* (%)	5 (45) adhesions	6 (86) tumors
	2 (18) incisional hernia
	2 (18) volvulus	1 (14) adhesions
	1 (9) tumor	
	1 (9) retroperitoneal neoplasm	

## Data Availability

The data used to support the findings of this study are included within the article. The additional data—i.e., the Excel database—used to support the findings of this study are available from the corresponding author upon request only as anonymized data.
